# Low-cost, LoRa GNSS tracker for wildlife monitoring

**DOI:** 10.1016/j.ohx.2025.e00669

**Published:** 2025-07-02

**Authors:** Adam F. Parlin, Ned A. Horning, Jason P. Alstad, Bradley J. Cosentino, James P. Gibbs

**Affiliations:** a Department of Fish, Wildlife, and Conservation Biology, Colorado State University, Fort Collins, CO, USA; b Wildlife Movement Institute, Savanah, GA, USA; c Department of Environmental Biology, State University of New York College of Environmental Science and Forestry, Syracuse, NY, USA; d Center for Biodiversity and Conservation, American Museum of Natural History, New York, NY, USA; e Department of Biology, Hobart and William Smith Colleges, Geneva, NY, USA

**Keywords:** Biologging, Animal movement, Low power, Conservation, Open source

## Abstract

The advent of GNSS tracking has allowed researchers to obtain detailed information on animal movement, which informs basic natural history and conservation management decisions. However, many devices are tailored to specific taxa thus limiting broader applicability. We present an open-source LoRaWAN (long range wide area network) GNSS (Global Navigation Satellite System) tracker, and an alternative commercial-off-the-shelf (COTS) development board global positioning system (GPS) tracker, a subset of the GNSS system. The COTS development board tracker provides a pathway for designing and implementing a general purpose LoRaWAN tracking unit, while the advantages of the Wildlife Movement Institute (WMI) tracker permit specific animal tracking and additional information to be collected, such as battery voltage, estimated precision error, and received signal strength intensity. Both units have documentation for setting up a LoRa application and network server and can be easily programmed using the Arduino Integrated Development Environment. To test the utility of these trackers in a LoRa data transmission application, we pilot tested the units on Eastern gray squirrels in Syracuse, New York, USA. Our trackers highlight the capability for customizable, open-source tracking technology that can be tailored to a suite of study organisms allowing researchers to design, develop, and deploy low-cost, specialized wildlife tracking equipment.

## Graphical abstract

**Figure:**
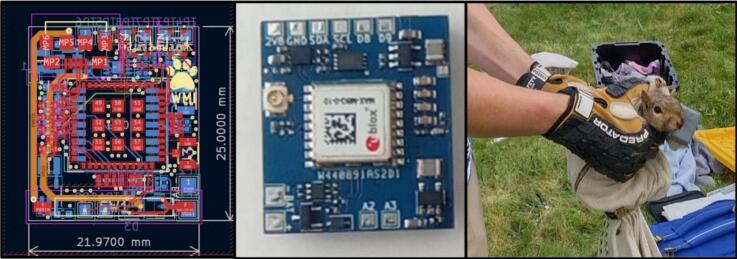

**Table:** 

**Specifications table**	
**Hardware name**	• Wildlife Movement Institute (WMI) LoRa Tracker
**Subject area**	• Conservation • Biologging • Ecology
**Hardware type**	• Monitoring wildlife location • Environmental monitoring
**Closest commercial analog**	Gnat Asset Tracker (Tlera Corp.)
**Open-source license**	*CC BY 4.0* (Hardware) *MIT License* (Software)
**Cost of hardware**	COTS Development Board LoRa Tracker = $63 (per unit) WMI LoRa Tracker = $80 (per unit, >5 ordered at a time)
**Source file repository**	https://doi.org/10.17605/OSF.IO/JUE5X
**OSHWA certification UID** *(OPTIONAL)*	

## 1 Hardware in context

Biotelemetry is an important tool for monitoring animals under natural conditions. Wildlife tracking benefited from the use of the very high frequency (VHF) tracking units in the early 1960 s [1,2] where researchers would track radio signals either on the ground or through aerial surveys. By the early 1990 s, researchers were using global positioning system (GPS) based telemetry systems to monitor fine-scale movement of wildlife [3]. The recent rise in open-source technology, such as the Arduino hardware and software platform [4], has led to a rapid increase in the design, development, and deployment of newer, smaller tracking technologies [5]. However, many biologists are limited in their capacity to design tracking equipment and therefore must purchase proprietary tracking units that can cost an order of magnitude more than building from open-source materials.

The components behind a Global Navigation Satellite System (GNSS) tracking unit remain relatively similar across devices [6,7], with primary differences including the attachment method on the study organism of interest and whether the data are logged or reported in near real time. For instance, many trackers will use GNSS module (e.g., uBlox; Zürcherstrasse, CH) as the base for obtaining location data, and save the data to an onboard microcontroller unit. As a result, these data must be either downloaded directly after recapturing the study animal, or through peer-to-peer communication with a base station style receiver, which requires obtaining a relative location of the animal and being within a necessary distance for data transfer.

LoRa (Long Range), the physical radio technology that enables long-range and low-power communication, and LoRaWAN (LoRa wide area network), the network protocol for managing communication between devices and the network, provide an opportunity to remotely collect data from a tracker unit without having to recapture the animal or determine the approximate location. Unlike peer-to-peer technologies like Bluetooth (<1000 m), LoRaWAN (>1000 m) communicates through gateways, thus making it suitable for wide-area applications [8]. LoRaWAN technology consists of both a global network and private networks made of base stations where units manage data packets and transmit a few bytes of sensor data (uplink) or receive short commands (downlink) before entering a low power state; further, LoRaWAN has been proven to be a valuable tool in wildlife research [5]. While the learning curve may be steep, open-source technologies facilitate learning, innovation, and development of future technologies [9].

## 2 Hardware description

Here we describe two forms of an open-source LoRaWAN tracker. The first is a custom-built LoRaWAN GPS tracker built using commercial-off-the-shelf (COTS) components including the Arduino Pro Mini and additional breakout board components that can help facilitate the learning of design, development, and deployment. The second is a fabricated LoRa GNSS tracker based on a modified version of the GNAT asset tracker (https://www.tindie.com/products/tleracorp/gnat-loragnss-asset-tracker/). Both units are capable of transmitting data to a LoRa gateway, where those data are then uploaded to a server and then database for viewing. The Gnat asset tracker can be used as an alternative to the WMI LoRa tracker, but the hardware cannot be modified by the user.

In addition to facilitating the learning of design considerations for a LoRa-based tracker, the COTS development board tracker, hereafter COTS LoRa tracker, also served as a contrast with the WMI LoRa tracker in terms of size and power consumption. With the GNSS/GPS patch sitting on top of the board and no enclosure or battery, the COTS LoRa tracker was approximately 38 x 29.5 15 mm (LxWxH) while the WMI tracker was 22 x 25 x 11 mm (LxWxH). The COTS LoRa tracker had a power draw around 100 mA during GPS sampling, 7 mA in between LoRa transmission attempts (each attempt drew 150 mA and lasted 30 ms), and a 500uA draw in a low power state. In contrast, the WMI LoRa tracker had a power draw around 40 mA during GNSS sampling, 7 mA in between LoRa transmission attempts, and < 10uA in low power state. Power measurements were completed with a Power Profiler Kit II (nRF-PPK2; Nordic Semiconductor, Trondheim, NO).

In a field research setting, these tracking units allow researchers to:

• Monitor smaller wildlife (>500 g) with remote data transmission to obtain up-to-date information.
• Customize the enclosure, such as 3D printing or encapsulation with epoxy, to allow for species-specific fit ranging from collar design to glue-on methods.
• Modify the PCB board to include additional sensors for data transmission (e.g., photosensor, humidity, pressure, etc.), triggers to change sampling parameters (e.g., increased location recording during intense activity), or identify events on-board (e.g., active, inactive, or mortality states).

## 3 Design files summary

**Table:** 

**Design filename**	**File type**	**Open source license**	**Location of the file**
Gerber_files	.zip	*CC BY 4.0*	https://doi.org/10.17605/OSF.IO/JUE5X
Pick_and_place_files	.zip	*CC BY 4.0*	https://doi.org/10.17605/OSF.IO/JUE5X
3D_step_file	CAD file (.step)	*CC BY 4.0*	https://doi.org/10.17605/OSF.IO/JUE5X
Bill_of_materials	excel spreadsheet (.csv)	*CC BY 4.0*	https://doi.org/10.17605/OSF.IO/JUE5X
WMI_LoRa_Tracker_Design_Files	.zip (.kicad_sch)	*CC BY 4.0*	https://doi.org/10.17605/OSF.IO/JUE5X
Component_cost	.csv	*CC BY 4.0*	https://doi.org/10.17605/OSF.IO/JUE5X
COTS LoRa Tracker	Arduino Sketch (.ino)	*Non-commercial license: CC BY-NC-ND 4.0*	https://doi.org/10.17605/OSF.IO/JUE5X
WMI_LoRa_Tracker	Arduino Sketch (.ino)	*Non-commercial license: CC BY-NC-ND 4.0*	https://doi.org/10.17605/OSF.IO/JUE5X

For production of the WMI tracker, the *Gerber File*, *Pick_and_Place_files*, and *Bill_of_materials* can be used to order the boards. The *Gerber_files* contain the design files for the production of the PCB. The *Pick_and_Place_files* contain information necessary to place components onto the board using a machine for assembly. To ensure the appropriate components are purchased, the *Bill_of_Materials* is included and can be uploaded when ordering boards. The *Component_cost* file shows a breakdown of the cost per part for the WMI LoRa Tracker. To facilitate design of an enclosure, the *3D_step_file* includes a 3D CAD (computer aided design) model that can be uploaded into CAD software for design (e.g., Autodesk Fusion 360; Autodesk, Inc., San Francisco, CA, USA). For the WMI LoRa Tracker, the design schematics can be found in the *WMI_LoRa_Tracker_Design_Files*, which can be modified based on the needs of the user. Finally, the *COTS_LoRa_Tracker* file contains the Arduino sketch (.ino) necessary to program the tracker, while the *WMI_LoRa_Tracker* file contains the Arduino sketch necessary to program the WMI Tracker.

### 3.1 Bill of materials

The bill of materials for the WMI LoRa tracker can be found in the Open Science Framework (OSF) repository under the *Bill_of_materials* file, and a breakdown of individual component costs under the *Component_cost* file. The COTS LoRa tracker parts (Table 1) are for the hardware components and may require additional parts depending on the needs of the researcher. The uFL antenna (915 MHz) is used for both the WMI LoRa tracker and the COTS LoRa tracker, while the RF antenna GPS ceramic patch is only necessary for the WMI LoRa Tracker.

**Table 1:** Components required for manual assembly. All parts can be purchased through Digikey (https://www.digikey.com) based on the part number. The first six components are required for the COTS LoRa tracker, while the RF antenna GPS ceramic patch is required for the WMI LoRa tracker. Both trackers use the same uFL whip antenna (915 MHz).

**Component**	**Tracker Unit**	**Number**	**Cost per unit − cur- rency**	**Total cost − currency**	**Source of materials**	**Part Number**
Arduino Pro Mini	COTS LoRa Tracker	1	$11 USD	$11 USD	Digikey	1568–11114-ND
RFM95W Breakout Board	COTS LoRa Tracker	1	$20 USD	$20 USD	Digikey	1528–1667-ND
GPS Module (GP-20U7 or GP-181MK)	COTS LoRa Tracker	1	$20 USD	$20 USD	Digikey	1568–19166-ND
NPN Transistor (PN2222)	COTS LoRa Tracker	1	$0.50 USD	$0.50 USD	Digikey	PN2222AD26ZCT-ND
uFL Antenna (915 MHz)	COTS/WMI LoRa Tracker	1	$10 USD	$10 USD	Digikey	1173–1135-ND
uFL Connector Jack	COTS LoRa Tracker	1	$0.80 USD	$0.80 USD	Digikey	343-CONMHF1-SMD-G-TCT-ND
RF ANTENNA GPS Ceramic Patch	WMI LoRa Tracker	1	$7.50 USD	$7.50 USD	Digikey	ACTPAT182-01-IP

## 4 Bill of materials summary

For assembly of the WMI LoRa tracker, certain parts may require a minimum order quantity. For instance, R10 (ARG02DTC2702) and R11 (0402WGD1003TCE) in the *Component_cost* file require a minimum-order-quantity of 10000. As of 2024, through the PCBWay assembly service (https://www.pcbway.com/), these components are basic parts and are priced according to the number required. The estimated cost of the WMI LoRa tracker is based on an order of 5 units, which splits the assembly cost to approximately $80 per unit. As of 2024, the assembly cost for all 5 units was $29 USD. Components and manufacturer part number for the COTS LoRa Tracker can be found in Table 1. The GPS module for the COTS LoRa tracker can use either the GP-20U7 or the GP-181MK depending on availability as they are interchangeable. Other costs can include materials for designing enclosures, such as 3D printer filament or epoxy resin for encapsulation. Note that the COTS and WMI LoRa trackers were tested in the USA, which permits LoRa data transmission on the 915-MHz (902–928 MHz) industrial, scientific, and medical (ISM) bands. Researchers should check appropriate frequency bands for LoRa and adhere to their own country’s regulations.

## 5 Build instructions

### 5.1 Setting up a LoRaWAN ChirpStack server

Unless you have access to an internet server, we recommend using a virtual machine, such as DigitalOcean (DigitalOcean Holdings, Inc; Ney York City, NY, USA), to run the necessary servers for the LoRaWAN. The virtual machine permits customizing the ChirpStack application and network servers necessary for receiving transmitted data and storing in databases. Alternatives include using The Things Network (TTN) and connecting a public gateway to utilize a broader network of receiving towers. Note that there are limitations on data transmission when using TTN for fair use by the community, such as an average of 30 s uplink time on air per 24 h per device and at most 10 downlink messages per 24 h [10,11]. For the COTS LoRa tracker, the cayenneLLP is used to decode the data. For the WMI LoRa tracker, an optional codec is provided increase the precision of the coordinates. We provide detailed instructions regarding setup through the WMI GitHub repository [https://github.com/WildlifeMovement].

*General safety:* When possible, purchase batteries with solderable tabs. Avoid high heat contact with batteries as this will reduce the lifespan of the battery.

### 5.2 COTS LoRa tracker

**Fig. 1:**
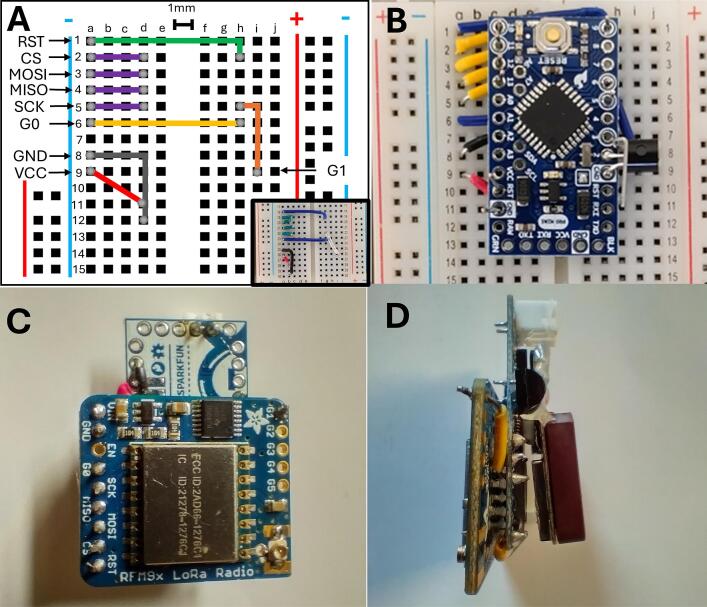
**Assembly of the COTS development board tracker**. (A) Breadboard configuration or 22AWG wires for connecting to the Arduino Pro Mini. (B) Example of Arduino pro mini fitted to the wire connections. It is recommended to upload the Arduino sketch prior to assembly. (C) connection of the RFM95W breakout board to the Arduino pro mini. (D) Side view of the tracking unit with the GPS unit connected, and a JST connection for power after programming.

Note: For breadboard prototyping, it is recommended to start with jumper cables. The instructions below are for making the unit compact and ready for deployment (Fig. 1).

**Fig. 2:**
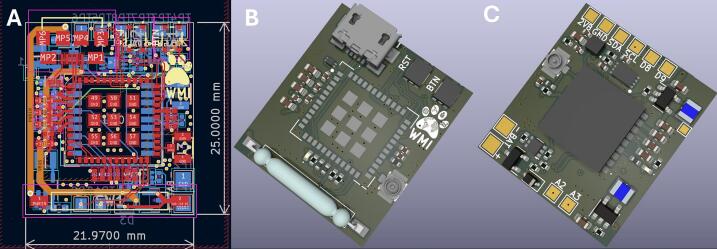
**Configuration of the GNSS Tracker**. WMI LoRa tracker (A) size and CAD images of the (B) front and (C) back of the unit. The reed switch is a normally-open (NO) switch and assembly of a case should take into consideration the need to apply a magnet to turn the unit on. Users can omit the placement of the reed switch during production and attach their own, modified NO switch.
• Part C: Combining the Pro Mini and RFM95W LoRa module
  1. On a breadboard, you will cut the hookup wires so that the Pro Mini fits onto the hookup wires, and when the board is removed it can be fit into the RFM95W module.
  2. Starting at the top of the board, cut the hookup wires so that the following wires fit into the respective letter-number combinations. Note: distance between each terminal is approximately 1 mm.
  3. When complete, fit the Pro Mini into the hookup wires on the board and solder each connection.
• Part D: Adding the GPS receiver
  1. Cut the wires on the GPS unit to the appropriate length for your final design.
  2. Solder the black wire (GND) to the collector on the NPN transistor.
  3. Solder the white wire (Tx) to the Pin 4 on the Pro Mini.
  4. Solder the red wire (Power) to the VCC on the Pro Mini.
    a. It is helpful to have extra length to the hookup wire sticking through, so it is easier to solder the power together without causing a bridge between VCC and GND.

• Part A: Soldering the NPN Transistor
  1. Begin by first soldering the NPN transistor to the Arduino Pro Mini (hereafter, ‘Pro Mini’). First, fit the emitter (GND) and base (Pin 2) through the holes on the Pro Mini, and bend the transistor so the flat side is level with the board, approximately a 90-degree angle. Solder the emitter to the GND, and the base to the Pin 2 on the Pro Mini. Bend the collector to prepare soldering the GND from the GPS unit to the transistor.
• Part B: Preparing the RFM95W LoRa Module
  1. First, solder the uFL connector jack to the RFM95W breakout board.
    a. To combine the components into a single, compact unit, 22AWG hookup wires need to be cut to specific lengths and bent at 90-degree angles to allow connection of the boards (Fig. 2).

### 5.3 WMI LoRa tracker

Part A. Ordering the WMI LoRa Tracker

Note: These instructions are generalized for PCBWay as of August 2024. The WMI LoRa tracker (Fig. 2) can be modified as needed by the user. Additionally, the current tracker uses a NO (normally-open) reed switch. A smaller magnet will need to be fixed to the switch to ensure the unit turns off when a magnet is applied.

1. Begin by going under the PCB instant quote and locate the ‘Quick order PCB.’
2. Add the Gerber file (as a .ZIP) under the PCB specification section. This should detect the number of layers (4 layers) and board size (22 x 25 mm) (Fig. 2).
3. Go under ‘Assembly Service’ to have the company assemble the tracker unit for you, otherwise you will only receive a PCB with no components.
4. For assembly, add the following additional files:
  a. Parts List (BOM): add the Bill_of_materials.csv file.
  b. Upload Centroid file: add the Pick_and_place_files.zip folder
5. Complete the order.

## 6 Operation instructions

### 6.1 Prior to deployment

After setting up and testing the ChirpStack application and network server, the trackers are ready to be tested with the server to ensure data transmission are at the desired sampling frequency. The code outlined here focuses on the tracker calculating GPS coordinates, and then immediately sending the data packet to the nearest gateway. These codes can be modified to the desired specifications of the user, such as storing the data on the EEPROM, or changing the conditions for when coordinates should be obtained (e.g., diurnal vs. nocturnal, etc.).

### 6.2 Programming the tracking units

Programming the COTS LoRa tracker will require a USB-FTDI converter to program the pins. The WMI LoRa tracker can be programmed using a micro-USB and will require setup of the ArduinoCore-stm32l0 library [12]. Both units can be coded using the Arduino IDE. Below we first outline the COTS LoRa tracker code then the WMI LoRa Tracker (https://osf.io/jue5x/).

### 6.3 COTS LoRa tracker

The following Arduino libraries are required to run the code:

Arduino-LMIC [13]; [https://github.com/mcci-catena/arduino-lmic], Low-Power [14]; [https://github.com/rocketscream/Low-Power], ellapsedMillis [15]; [https://github.com/pfeerick/elapsedMillis], TinyGPSPlus [16]; [https://github.com/mikalhart/TinyGPSPlus], CayanneLLP [17]; [https://github.com/ElectronicCats/CayenneLPP].

The beginning portion of the code uses little endian byte order (least significant byte, LSB first) for the device EUI, and application key. The Application EUI is not required. Then, the pin map needs to be defined to connect the Pro Mini with the RFM95 LoRa module. The variables for sampling intervals can be programmed to change how long the device stays on, how long the unit will search for a GPS signal, and preparation for the data packet. When the code is uploaded, and upon setup, the NPN transistor is in an ‘Off’ state to avoid power drain. Upon each attempt, the tracker will first turn on and obtain coordinates. If no coordinates are obtained, then the tracker will send zeros in place of latitude and longitude. If the unit obtains coordinates, the remainder of the time will be spent connecting to the nearest LoRa gateway to transmit the data packet with coordinates. The latter part of the code sets the sleep interval for how long until the next attempt to obtain coordinates and transmit to the nearest gateway.

### 6.4 WMI LoRa tracker

The libraries required for the WMI Tracker are found compiled within the ArduinoCore-stm32l0 folder when downloaded. The following header files are used in the code: STM32L0.h, EEPROM.h, LoRaWAN.h, TimerMillis.h, GNSS.h, RTC.h.

The GNSS tracker uses the following board manager, ArduinoCore-stm32l0 [12], to permit uploading code through the Arduino IDE onto the unit. Instructions for installing the necessary drivers and STM32 Bootloader are documented on the GitHub (https://github.com/GrumpyOldPizza/ArduinoCore-stm32l0).

The simple tracker code initializes the LoRaWAN, real-time clock (RTC), GNSS modules, and configures necessary settings to monitor battery voltage as an indicator of tracker status. The LoRaWAN initialization retrieves the device EUI and configures the appropriate settings (frequency plan, data rate, transmission power, sub-band). The device then joins the LoRaWAN network using the OTAA (over-the-air-activation) and sets up a callback for receiving data if user wants to change settings when the unit is deployed. The battery monitoring setup configures pins to monitor voltage and sets the analog-to-digital converter (ADC) resolution. It is important to note that temperature will cause changes in the voltage, but a considerable drop can indicate when the battery is near depletion. The unit does not have a RTC, but instead uses an initial dummy date and time and uses the GNSS time after first coordinate fix. The GNSS configuration sets the satellite constellations (i.e., 11 = GPS + GLONASS + Galileo; 13 = GPS + Beidou + Galileo) and selects the external antenna.

Within the loop, the unit will handle locations, and subsequently enter a stop mode to save power. During the GNSS handling, the unit will check if location data are available during the myAcqTime period. If a valid fix is obtained, it will calculate the latitude, longitude, and EPE, and calls the GetCoords() function to process the coordinates.

### 6.5 Additional LoRa parameters to record

Within the ChirpStack server researchers can record additional LoRa parameters specific to trackers, including packets received, errors, signal-to-noise ratio (SNR), and received signal strength intensity (RSSI). Researchers can also obtain information on the gateway, or gateways, that received the packet, permitting checks on the line-of-sight transmission for gateway placement. These data would have to be integrated into the database collection by the individual.

## 7 Validation and characterization

### 7.1 Proof-of-concept with eastern gray squirrels

**Fig. 3:**
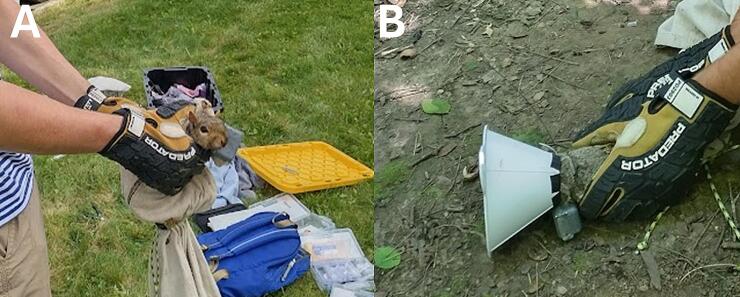
**Deployment of trackers**. (A) Front and (B) profile view of the collar attachment on eastern gray squirrels. Handling time, which included removing the animal from the cage, weighing, and attaching the collar, was under < 5 min. Handling and attachment of collars followed SUNY-ESF IACUC protocol #200801, and devices were < 5 % of the animal’s mass.

During June and July 2023, we tested our WMI tracker (n = 2) and Arduino COTS tracker (n = 2) on an Eastern gray squirrel (*Sciurus carolinensis*) in Syracuse, New York, USA. We limited the monitoring period to five days to ensure we could recapture individuals and retrieve the devices. Data collected after the fifth day was not included in the comparison. After capture, the squirrels were coaxed into an opaque bull denim bag and weighed to ensure units did not exceed 5 % of the body mass. A zipper allowed the head of the squirrel to come out, at which point a flexible cone was placed around the neck to limit the vision of the animal and reduce stress (Fig. 3) [18]. Once the collar was attached, the cone was then removed and the squirrel released. This approach did not require an anesthetic and resulted in no mortalities. The COTS LoRa trackers were programmed to record every hour prior to assembly. The WMI LoRa trackers can be easily programmed after assembly and during this pilot test we set the units to record every 15 min. LoRaWAN gateways (Dragino DLOS8N – 915 MHz; approx. $390 USD/gateway in 2022; Dragino Technology, Shenzhen, CHN) were placed at several locations, with the highest elevation at Skytop (N43.01, W76.12; elevation 187 m). We monitored squirrels at Westminster Park (43.03, −76.11; elevation = 171 m) and Thornden Park (43.04, −76.12; elevation = 172 m). In addition to the tracker, a refurbished VHF transmitter (BD-52, Holohil; Carp, ON, CAN) was attached to permit locating the individual for recapture. Total weight of the collar was < 5 % of the animal’s body weight. Handling and attachment of collars followed SUNY-ESF IACUC protocol #200801.

**Fig. 4:**
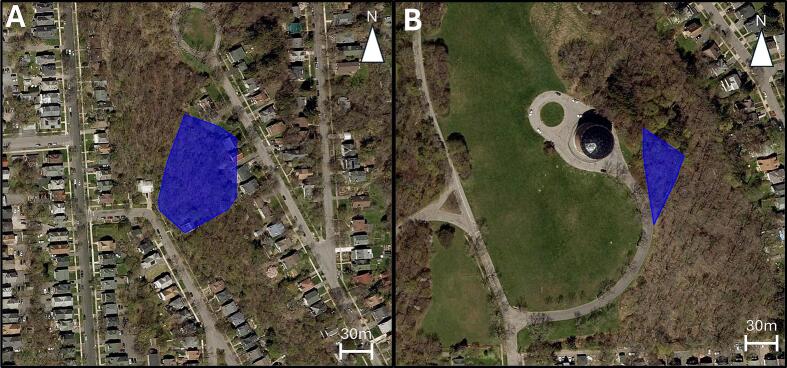
**Squirrels remotely monitored with COTS LoRa tracker.** Home range estimates (95 % MCP) for eastern gray squirrels over an approximate 5-day monitoring period using the COTS LoRa tracker unit. The (A) male squirrel had 105 fixes, and the (B) female squirrel had 112 fixes. Data transmission was received by a gateway (A) 2.6 km and (B) 3.2 km from the monitoring location.

**Fig. 5:**
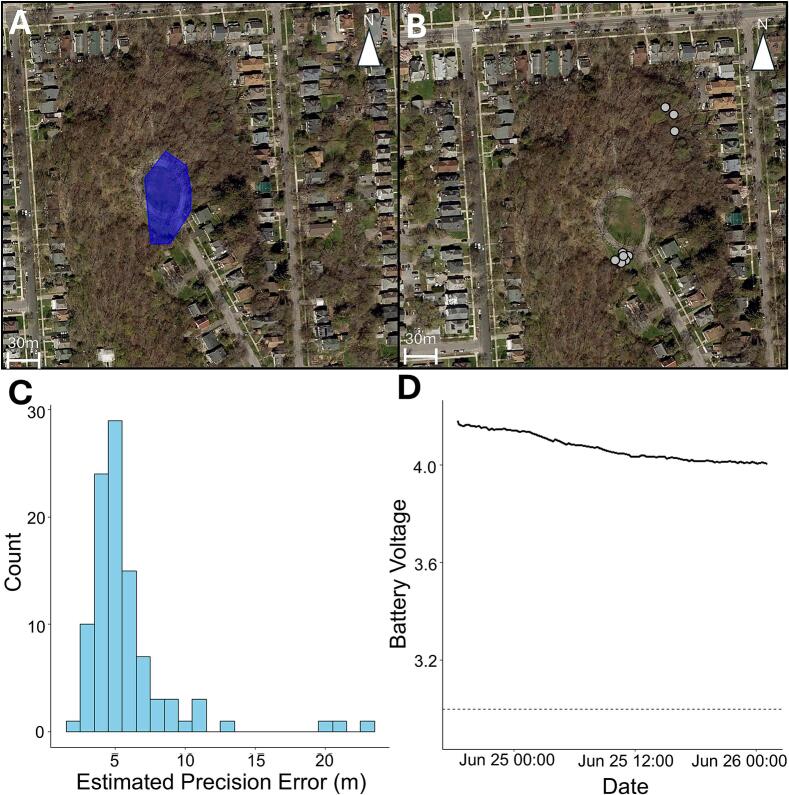
**Squirrels remotely monitored with WMI LoRa tracker**. (A) Home range estimate (95 % MCP) and (B) location data, represented with a grey circle and black stroke, for eastern gray squirrels monitored using the WMI tracker. One left the study site, the signal went missing, and we were unable to cover the tracking unit. For the individual we were able to recover, the (C) estimated precision error showed that a vast majority of recordings (93 %) were within ± 10 m. The battery voltage (D) indicated that the battery was fully charged and well above the threshold for when the unit would stop transmitting data (dashed line). The data transmission was received by a gateway 2.6 km from the study location.

We generated a minimal convex polygon (95 % MCP) using the *adehabitatHR* R package [19] to show the area where the coordinates were collected for each individual squirrel. Given that there was a limited tracking period, the MCP is more representative of an area occupied than a true home range. The trackers were able to transmit data 2.6 km (Westminster Park) and 3.2 km (Thornden Park) to the farthest gateway (Fig. 4; Fig. 5). One individual went missing shortly after deployment (Fig. 5B) and we were unable to recover the unit or locate the device using the VHF transmitter at the study site or driving around the city. In this study system, it is not uncommon for individuals to vanish as they are prone to road mortality and predation [20], which can result in destruction of the device (road mortality) or displacement by avian and mammalian predators. While mortality and device destruction are unavoidable, mitigation related to animal behavior (e.g., transient movement outside of LoRa gateways) can be done. For instance, modifications to the Arduino sketch could include saving the location to the EEPROM when outside of the network’s range and then sending multiple transmissions when the animal returns. The WMI LoRa tracker was able to transmit the estimated precision error (EPE; Fig. 5C) and battery voltage (Fig. 5D), while the COTS LoRa tracker was unable to report battery voltage and did not obtain EPE in the code.

### 7.2 Battery chemistry considerations and power consumption

**Fig. 6:**
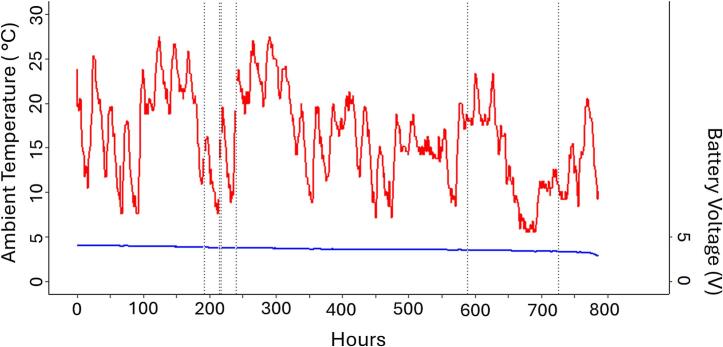
**Complete power drain of a LiPo battery on a WMI Tracker with corresponding ambient temperature.** The unit had a 400 mAh power supply (3.7v LiPo battery) and was programmed to record a GNSS location every hour at a fixed location outside along a hedgerow. The fully charged battery recorded a coordinate every hour for 34 days (782 recordings, 6 missed packets; packet loss = 0.7 %) until the voltage dipped below 3.0 V after 750 h and the unit then stopped. Ambient temperatures during this time fluctuated between 7.5 – 27.5 °C. Black vertical dashed lines indicate packet loss and missed GNSS recordings. Ambient temperatures recorded at the unit’s location are red, and the battery voltage recorded is blue. (For interpretation of the references to colour in this figure legend, the reader is referred to the web version of this article.)

The choice of battery chemistry and size is dependent on the constraints of the study system, including frequency of sampling, total weight of the device (< 5 % of animal mass), and how long to monitor the animal. Our selection of 3.7 V LiPo batteries (PRT-13851; Sparkfun, CO, USA) in the proof-of-concept was for flexibility in reusing devices. Energy dense batteries (e.g., lithium thionyl chloride, LiSOCl_2_) are possible to use with the trackers. However, there is a voltage spike when the unit begins LoRa transmission that can cause issues with the devices. The WMI LoRa tracker has an added buck/boost converter on the board, while we added a supercapacitor to the COTS LoRa tracker when using LiSOCl_2_ chemistry. Our recommendation for the WMI LoRa tracker over the COTS LoRa tracker is in part due to the energy efficiency during sampling. A brief test with the WMI LoRa tracker in a stationary location using a 400 mAh 3.7v LiPo battery showed that the unit can record GPS coordinates every hour for up to 34 days (Fig. 6) with a package loss rate of 0.7 %. A separate test over 31 days had a loss rate of 1.06 % (8 missed out of 752 total recordings). Future considerations for boards could include solar recharging to increase the longevity of monitoring animals of interest.

### 7.3 Considerations of LoRa-based trackers

It is important to keep in mind that the LoRa data transmission operates via line-of-sight. During our proof of concept using grey squirrels, we had an individual that disappeared from the study site. While the likely cause is road related mortality due to the VHF signal disappearing along with transmitted coordinates, there is the possibility that the animal left the study site altogether. These behavioral shifts can be considered when writing the code for data collection and transmission, such as saving the data on-board (e.g., EEPROM) and subsequently transmitting the data when it is back in range of the LoRa gateway. The placement of gateways can also be part of experimental design, whereby a few gateways are placed outside of the study area to capture these types of long-range movements.

The LoRa tracking device that went missing cost under $100 USD. In contrast, proprietary equipment can significantly more (e.g., >$1000/tracker) and may require additional funds for data transmission [21]. In this study system, where individual mortality can be high [20], the advantage of utilizing open-source technology like the WMI LoRa tracker enables adequate data collection in longer-term monitoring efforts and the possibility of determining the fate of individuals. Ten WMI LoRa trackers could be deployed instead of one hypothetical proprietary tracker thus buffering against unavoidable causes of mortality. Researchers must consider the constraints of their study system when choosing a tracking device; however, being able to deploy more units is advantageous for data collection and ensuring project success.

## Ethics statements

Field monitoring of eastern gray squirrels (*S. carolinensis*) followed SUNY-ESF IACUC protocol 200,801 and NYSDEC LCP SCI permit 3049.

## CRediT authorship contribution statement

**Adam F. Parlin:** Writing – review & editing, Writing – original draft, Visualization, Validation, Software, Resources, Project administration, Methodology, Investigation, Formal analysis, Data curation, Conceptualization. **Ned A. Horning:** Writing – review & editing, Software, Methodology, Funding acquisition, Data curation, Conceptualization. **Jason P. Alstad:** Writing – review & editing, Funding acquisition, Conceptualization. **Bradley J. Cosentino:** Writing – review & editing, Funding acquisition, Conceptualization. **James P. Gibbs:** Writing – review & editing, Funding acquisition, Conceptualization.

## Declaration of competing interest

The authors declare that they have no known competing financial interests or personal relationships that could have appeared to influence the work reported in this paper.
